# (*E*)-3-(4-Chloro­phen­yl)-3-[3-(4-chloro­phen­yl)-1*H*-pyrazol-1-yl]prop-2-enal

**DOI:** 10.1107/S1600536810035646

**Published:** 2010-09-18

**Authors:** V. Susindran, S. Athimoolam, S. Asath Bahadur, R. Manikannan, S. Muthusubramanian

**Affiliations:** a Department of Lighthouses & Lightships, Ministry of Shipping, Nagapattinam Lighthouse & DGPS station, Nagapattinam 611 001, India; b Department of Physics, University College of Engineering Nagercoil, Anna University Tirunelveli, Nagercoil 629 004, India; c Department of Physics, Kalasalingam University, Anand Nagar, Krishnan Koil 626 190, India; d Department of Organic Chemistry, Madurai Kamaraj University, Madurai 625 021, India

## Abstract

In the title compound, C_18_H_12_Cl_2_N_2_O, the pyrazole ring is almost planar [r.m.s. deviation = 0.002 Å] while the two chloro­phenyl rings are twisted out from the plane of the pyrazole ring, making dihedral angles of 5.3 (1) and 65.34 (4)°. In the crystal, centrosymmetric *R*_2_^2^(24) dimers are formed about crystallographic inversion centres through a pair of C—H⋯Cl inter­actions. These dimers are further linked through a C—H⋯O hydrogen bond, forming a *C*(8) chain extending along the *a* axis. C—H⋯π inter­actions are also observed.

## Related literature

For the pharmacological properties of pyrazoles and their derivatives, see: Baraldi *et al.* (1998 ▶); Bruno *et al.* (1990 ▶); Chen & Li (1998 ▶); Cottineau *et al.* (2002 ▶); Londershausen (1996 ▶); Mishra *et al.* (1998 ▶); Magedov *et al.* (2007 ▶); Rovnyak *et al.* (1982 ▶); Smith *et al.* (2001 ▶); Velaparthi *et al.* (2008 ▶); Wamhoff *et al.* (1993 ▶). For hybridization and electron delocalization around N atoms, see: Beddoes *et al.* (1986 ▶); Jin *et al.* (2004 ▶). For hydrogen bonding, see: Desiraju & Steiner (1999 ▶) and for hydrogen-bond motifs, see: Etter *et al.* (1990 ▶).
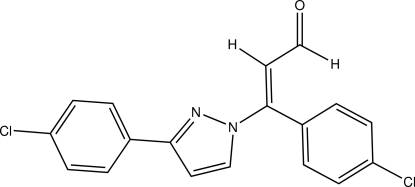


## Experimental

### 

#### Crystal data

C_18_H_12_Cl_2_N_2_O
*M* *_r_* = 343.20
Triclinic, 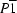

*a* = 9.4321 (6) Å
*b* = 9.6081 (5) Å
*c* = 9.9439 (7) Å
α = 90.533 (7)°
β = 116.924 (4)°
γ = 93.427 (6)°
*V* = 801.38 (9) Å^3^
*Z* = 2
Mo *K*α radiation
μ = 0.41 mm^−1^
*T* = 293 K
0.16 × 0.14 × 0.12 mm

#### Data collection

Bruker SMART APEX CCD area-detector diffractometer
Absorption correction: multi-scan (*SADABS*; Sheldrick, 2001 ▶) *T*_min_ = 0.884, *T*_max_ = 0.993
8950 measured reflections
3464 independent reflections
2982 reflections with *I* > 2σ(*I*)
*R*_int_ = 0.018

#### Refinement

*R*[*F* ^2^ > 2σ(*F* ^2^)] = 0.040
*wR*(*F* ^2^) = 0.116
*S* = 1.04
3464 reflections
211 parameters
H atoms treated by a mixture of independent and constrained refinement
Δρ_max_ = 0.20 e Å^−3^
Δρ_min_ = −0.24 e Å^−3^

### 

Data collection: *SMART* (Bruker, 2001 ▶); cell refinement: *SAINT* (Bruker, 2001 ▶); data reduction: *SAINT*; program(s) used to solve structure: *SHELXTL/PC* (Sheldrick, 2008 ▶); program(s) used to refine structure: *SHELXTL/PC*; molecular graphics: *PLATON* (Spek, 2009 ▶); software used to prepare material for publication: *SHELXTL/PC*.

## Supplementary Material

Crystal structure: contains datablocks global, I. DOI: 10.1107/S1600536810035646/fb2206sup1.cif

Structure factors: contains datablocks I. DOI: 10.1107/S1600536810035646/fb2206Isup2.hkl

Additional supplementary materials:  crystallographic information; 3D view; checkCIF report

## Figures and Tables

**Table 1:** Hydrogen-bond geometry (Å, °) *Cg*1 is the centroid of the C31–C36 ring.

*D*—H⋯*A*	*D*—H	H⋯*A*	*D*⋯*A*	*D*—H⋯*A*
C13—H13⋯Cl2^i^	0.93	2.93	3.6447 (16)	134
C14—H14⋯O1^ii^	0.93	2.49	3.407 (2)	167
C36—H36⋯Cl1^iii^	0.93	2.90	3.6334 (16)	137
C17—H17⋯*Cg*1^iv^	0.93	2.68	3.4919 (18)	147

Symmetry codes: (i) 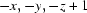; (ii) 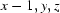; (iii) 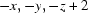; (iv) 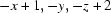.

**Fig. 1.**
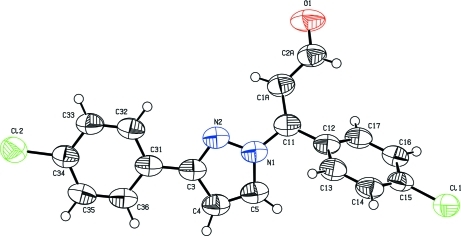
The title molecule with the atom numbering scheme. The displacement ellipsoids are shown at the 50% probability level.

**Fig. 2.**
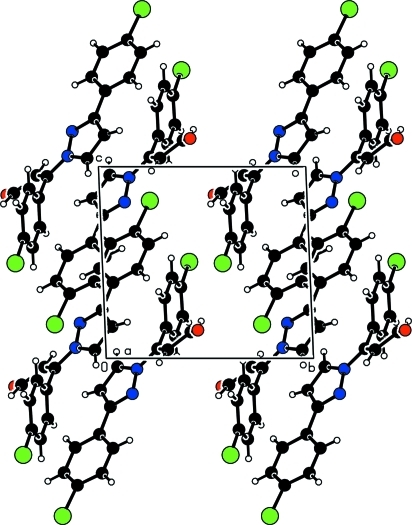
Packing diagram of the title structure viewed down the a axis. (Cl is shown in green, N in blue, O in red, C in black and H as a circle.)

**Fig. 3.**
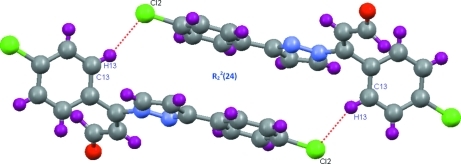
Ring R22(24) motif involving the C13—H13···Cl2 interactions. These interactions are drawn as dashed lines. (Cl is shown in green, N in blue, O in red, C in black and H in violet.)

**Fig. 4.**
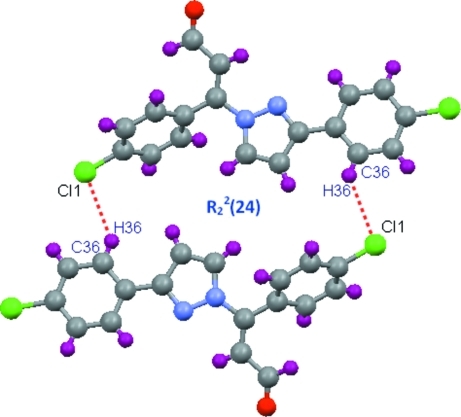
Ring R22(24) motif involving the C36—H36···Cl1 interactions. These interactions are drawn as dashed lines. (Cl is shown in green, N in blue, O in red, C in black and H in violet.)

**Fig. 5.**
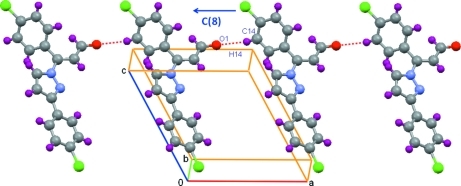
Chain C(8) motif involving the C14—H14···O1 hydrogen bond. The hydrogen bonds are drawn as dashed lines. (Cl is shown in green, N in blue, O in red, C in black and H in violet.)

**Fig. 6.**
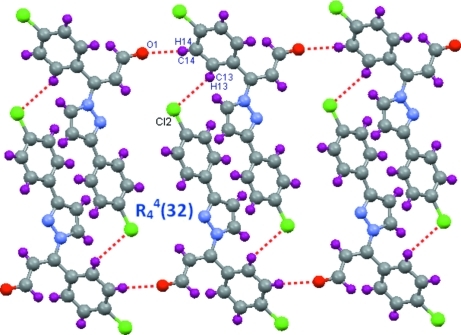
Secondary ring R44(32) motif formed by a combination of the ring R22(24) and the chain C(8) motifs. The C—H···O bonds and C—H···Cl interactions are drawn as dashed lines. (Cl is shown in green, N in blue, O in red, C in black and H in violet.)

## supplementary crystallographic information

### Comment

Pyrazole and its derivatives have been successfully tested for their fungicidal (Chen & Li, 1998), antihistaminic (Mishra *et al.*, 1998), anti-inflammatory (Smith *et al.*, 2001), antiarrhythmic and sedative (Bruno *et al.*, 1990), hypoglycemic (Cottineau *et al.*, 2002), antiviral (Baraldi *et al.*, 1998) activities. Pyrazole derivates possess antimicrobial (Velaparthi *et al.*,2008), anticancer (Magedov *et al.*, 2007) and anti-inflammatory (Rovnyak *et al.*, 1982) properties. They can also be used as biodegradable agrochemicals (Wamhoff *et al.*, 1993) as well as pesticides (Londershausen, 1996). Wide variety of biological effects of these molecules provoked interest for their crystal structure study and accordingly we have synthesized the title compound by multi-component reaction which conforms to principles of green chemistry. The crystal structure of the title compound is reported here.

The title molecule is shown in Fig. 1. The pyrazole ring is planar with the r.m.s. deviation equal to 0.002 Å. The sum of the bond angles at N1 of the pyrazole ring (359.7 (1)°) is in accordance with the *sp*^2^ hybridization of this atom (Beddoes *et al.*, 1986). The C—N bond lengths in the pyrazole ring are 1.370 (2) [C5-N1]  and 1.327 (2)Å  [C3-N2] long. These distances are shorter than the pertinent single bond length (1.443 Å), however, they are longer than the double bond length (1.269 Å) (Jin *et al.*, 2004). The values of these distances in the title structure indicate electron delocalization.

The two chlorophenyl rings are twisted out from the plane of the pyrazole ring with respective angles of 5.3 (1)° (C31//C36 ring) and 65.34 (4)° (C12//C17 ring). The propenal group assumes the extended conformation which is evidenced by the torsion angles of 173.3 (1)° [N1-C11-C1A-C2A] and 157.0 (2)° [C5-N1-C11-C1A]. The crystal structure is stabilized by intermolecular C—H···Cl, C—H···O and C—H···π-electron ring interactions (Tab. 1). The packing diagram of the title compound is shown in Fig. 2.

Each of two centrosymmetric *R*^2^_2_(24) dimers (Etter *et al.*, 1990) are formed around the crystallographic inversion centres  through a pair of the respective C—H···Cl interactions (Figs. 3 and 4 referring to C13—H13···Cl2 and C36—H36···Cl1, respectively; Tab. 1). Though the latter H···Cl distances are somewhat longer by about 0.5Å than the accepted values for  the C-H···Cl hydrogen bonding (Desiraju & Steiner, 1999) these dimers are an important motif in the present structure and therefore they are reported here.

These dimers are linked through a C—H···O bond making a chain C(8) motif (Etter *et al.*, 1990) that extends along the *a* axis of the unit cell (Fig. 5). Further, one of these primary ring *R*^2^_2_(24) motifs and the chain C(8) motif are combined to form a secondary ring *R*^4^_4_(32) motif (Fig. 6). Also, the C—H···π-electron ring interactions are observed in the structure. The crystallographic inversions link the latter motifs into pairs, forming another ring motif.

### Experimental

To a mixture of 1-(4-chlorophenyl)-1-ethanone N-[(*E*)-1-(4-chlorophenyl)ethylidene]hydrazone (0.003 mole) and 3 ml of dimethyl formamide kept in ice bath at 0°C, phosphorous oxycholride (0.024 mole) was added dropwise in 5 to 10 minutes. The reaction mixture was then irradiated under microwaves for 30 sec using a Biotage Microwave Synthesizer (frequency 2.45 GHz corresponding to the wavelength equal to 12.24 cm). The process of the reaction was monitored by thin layer chromatography using petroleum ether and ethyl acetate (4:1 v/v) as an eluent. The *R*_f_ value of the product was 0.62. After completion of the reaction, the reaction mixture was poured into crushed ice and extracted with dichloromethane. The organic layer was dried over anhydrous sodium sulfate. The title compound was separated by column chromatography: carrier: silica gel (60-120 mesh), eluent: petroleum ether and ethyl acetate mixture (98:2 v/v). The compound was crystallized from dichloromethane. Colourless crystals of a prismatic habitus with the average size of 0.5 × 0.5 × 0.2 cm were grown within a week.

### Refinement

All the H atoms were observable in the difference electron density map. All the hydrogens except the one from the aldehyde group were situated into the idealized positions and refined by the riding model approximation. The used values for the constraints: d(C—H) = 0.93 Å. *U*_iso_(H)= 1.2*U*_eq_(C). The positional parameters of the aldehyde hydrogen were refined freely while *U*_iso_(H)= 1.2*U*_eq_(C).

### Crystal data

**Table:**

C_18_H_12_Cl_2_N_2_O	*Z* = 2
*M**_r_* = 343.20	*F*(000) = 352
Triclinic, *P*1	*D*_x_ = 1.422 Mg m^−^^3^
Hall symbol: -P 1	Mo *K*α radiation, λ = 0.71073 Å
*a* = 9.4321 (6) Å	Cell parameters from 4879 reflections
*b* = 9.6081 (5) Å	θ = 2.4–24.7°
*c* = 9.9439 (7) Å	µ = 0.41 mm^−^^1^
α = 90.533 (7)°	*T* = 293 K
β = 116.924 (4)°	Block, colourless
γ = 93.427 (6)°	0.16 × 0.14 × 0.12 mm
*V* = 801.38 (9) Å^3^	

### Data collection

**Table:**

Bruker SMART APEX CCD area-detector diffractometer	3464 independent reflections
Radiation source: fine-focus sealed tube	2982 reflections with *I* > 2σ(*I*)
graphite	*R*_int_ = 0.018
ω scans	θ_max_ = 27.0°, θ_min_ = 2.1°
Absorption correction: multi-scan (*SADABS*; Sheldrick, 2001)	*h* = −12→11
*T*_min_ = 0.884, *T*_max_ = 0.993	*k* = −12→12
8950 measured reflections	*l* = −12→12

### Refinement

**Table:**

Refinement on *F*^2^	Primary atom site location: structure-invariant direct methods
Least-squares matrix: full	Secondary atom site location: difference Fourier map
*R*[*F*^2^ > 2σ(*F*^2^)] = 0.040	Hydrogen site location: difference Fourier map
*wR*(*F*^2^) = 0.116	H atoms treated by a mixture of independent and constrained refinement
*S* = 1.04	*w* = 1/[σ^2^(*F*_o_^2^) + (0.064*P*)^2^ + 0.1138*P*] where *P* = (*F*_o_^2^ + 2*F*_c_^2^)/3
3464 reflections	(Δ/σ)_max_ < 0.001
211 parameters	Δρ_max_ = 0.20 e Å^−^^3^
0 restraints	Δρ_min_ = −0.24 e Å^−^^3^

### Special details

**Table:**

Geometry. All esds (except the esd in the dihedral angle between two l.s. planes) are estimated using the full covariance matrix. The cell esds are taken into account individually in the estimation of esds in distances, angles and torsion angles; correlations between esds in cell parameters are only used when they are defined by crystal symmetry. An approximate (isotropic) treatment of cell esds is used for estimating esds involving l.s. planes.
Refinement. Refinement of *F*^2^ against ALL reflections. The weighted *R*-factor *wR* and goodness of fit *S* are based on *F*^2^, conventional *R*-factors *R* are based on *F*, with *F* set to zero for negative *F*^2^. The threshold expression of *F*^2^ > σ(*F*^2^) is used only for calculating *R*-factors(gt) *etc*. and is not relevant to the choice of reflections for refinement. *R*-factors based on *F*^2^ are statistically about twice as large as those based on *F*, and *R*- factors based on ALL data will be even larger.

### Fractional atomic coordinates and isotropic or equivalent isotropic displacement parameters (Å^2^)

**Table:**

	*x*	*y*	*z*	*U*_iso_*/*U*_eq_	
N1	0.32051 (15)	0.14597 (14)	0.94391 (16)	0.0637 (3)	
N2	0.33330 (15)	0.11932 (14)	0.81571 (16)	0.0645 (3)	
C3	0.23105 (17)	0.01018 (15)	0.74769 (19)	0.0609 (4)	
C4	0.1527 (2)	−0.03430 (17)	0.8341 (2)	0.0703 (4)	
H4	0.0765	−0.1085	0.8109	0.084*	
C5	0.2117 (2)	0.05315 (17)	0.9566 (2)	0.0704 (4)	
H5	0.1838	0.0510	1.0351	0.084*	
C11	0.42212 (17)	0.24992 (16)	1.05026 (18)	0.0616 (4)	
C12	0.36968 (16)	0.29488 (15)	1.16169 (18)	0.0588 (3)	
C13	0.21605 (17)	0.33822 (17)	1.1154 (2)	0.0656 (4)	
H13	0.1469	0.3408	1.0129	0.079*	
C14	0.16603 (17)	0.37703 (17)	1.2194 (2)	0.0674 (4)	
H14	0.0635	0.4053	1.1877	0.081*	
C15	0.26958 (18)	0.37363 (15)	1.3718 (2)	0.0617 (4)	
C16	0.42360 (18)	0.33354 (15)	1.42098 (19)	0.0625 (4)	
H16	0.4931	0.3333	1.5235	0.075*	
C17	0.47177 (16)	0.29416 (15)	1.31565 (18)	0.0604 (4)	
H17	0.5746	0.2665	1.3479	0.072*	
C31	0.21217 (16)	−0.04755 (15)	0.60370 (19)	0.0596 (4)	
C32	0.2953 (2)	0.01493 (17)	0.5314 (2)	0.0679 (4)	
H32	0.3586	0.0971	0.5730	0.082*	
C33	0.28550 (19)	−0.04239 (17)	0.4007 (2)	0.0695 (4)	
H33	0.3421	0.0002	0.3544	0.083*	
C34	0.19066 (18)	−0.16420 (17)	0.33797 (18)	0.0637 (4)	
C35	0.10245 (17)	−0.22597 (17)	0.4035 (2)	0.0670 (4)	
H35	0.0357	−0.3059	0.3590	0.080*	
C36	0.11458 (17)	−0.16782 (16)	0.5355 (2)	0.0651 (4)	
H36	0.0561	−0.2099	0.5803	0.078*	
C1A	0.55695 (18)	0.29870 (18)	1.0452 (2)	0.0694 (4)	
H1A	0.5853	0.2553	0.9778	0.083*	
C2A	0.65832 (19)	0.4138 (2)	1.1383 (2)	0.0739 (4)	
O1	0.78899 (15)	0.44765 (18)	1.14726 (18)	0.0993 (5)	
Cl1	0.20652 (6)	0.41935 (5)	1.50378 (6)	0.08373 (18)	
Cl2	0.18660 (6)	−0.24132 (5)	0.17751 (5)	0.08459 (18)	
H2A	0.612 (3)	0.475 (2)	1.198 (3)	0.102*	

### Atomic displacement parameters (Å^2^)

**Table:**

	*U*^11^	*U*^22^	*U*^33^	*U*^12^	*U*^13^	*U*^23^
N1	0.0550 (7)	0.0618 (7)	0.0774 (8)	0.0047 (6)	0.0326 (6)	0.0104 (6)
N2	0.0554 (7)	0.0635 (7)	0.0762 (8)	0.0042 (6)	0.0312 (6)	0.0107 (6)
C3	0.0500 (7)	0.0530 (7)	0.0810 (10)	0.0102 (6)	0.0297 (7)	0.0162 (7)
C4	0.0665 (9)	0.0589 (8)	0.0926 (12)	0.0006 (7)	0.0427 (9)	0.0115 (8)
C5	0.0654 (9)	0.0641 (9)	0.0900 (11)	0.0033 (7)	0.0426 (9)	0.0148 (8)
C11	0.0475 (7)	0.0620 (8)	0.0726 (9)	0.0099 (6)	0.0240 (7)	0.0146 (7)
C12	0.0444 (7)	0.0553 (7)	0.0743 (9)	0.0047 (6)	0.0247 (6)	0.0127 (7)
C13	0.0443 (7)	0.0698 (9)	0.0745 (9)	0.0073 (6)	0.0194 (7)	0.0093 (7)
C14	0.0446 (7)	0.0643 (9)	0.0910 (11)	0.0056 (6)	0.0285 (8)	0.0058 (8)
C15	0.0550 (8)	0.0488 (7)	0.0830 (10)	−0.0070 (6)	0.0341 (7)	0.0005 (7)
C16	0.0532 (8)	0.0544 (7)	0.0722 (9)	−0.0030 (6)	0.0225 (7)	0.0081 (7)
C17	0.0432 (7)	0.0573 (8)	0.0747 (9)	0.0061 (6)	0.0211 (6)	0.0139 (7)
C31	0.0450 (7)	0.0536 (7)	0.0775 (9)	0.0098 (6)	0.0246 (7)	0.0167 (7)
C32	0.0617 (9)	0.0571 (8)	0.0812 (10)	−0.0050 (7)	0.0300 (8)	0.0110 (7)
C33	0.0612 (9)	0.0686 (9)	0.0755 (10)	−0.0059 (7)	0.0293 (8)	0.0144 (8)
C34	0.0489 (7)	0.0652 (8)	0.0669 (9)	0.0057 (6)	0.0171 (6)	0.0132 (7)
C35	0.0483 (7)	0.0608 (8)	0.0811 (10)	−0.0025 (6)	0.0205 (7)	0.0100 (7)
C36	0.0477 (7)	0.0605 (8)	0.0856 (11)	0.0009 (6)	0.0290 (7)	0.0138 (8)
C1A	0.0498 (8)	0.0802 (10)	0.0790 (10)	0.0084 (7)	0.0292 (7)	0.0104 (8)
C2A	0.0507 (8)	0.0877 (11)	0.0792 (11)	0.0029 (8)	0.0260 (8)	0.0168 (9)
O1	0.0524 (7)	0.1234 (12)	0.1192 (12)	−0.0071 (7)	0.0379 (7)	0.0170 (9)
Cl1	0.0797 (3)	0.0800 (3)	0.1033 (4)	−0.0142 (2)	0.0546 (3)	−0.0152 (2)
Cl2	0.0784 (3)	0.0921 (3)	0.0749 (3)	−0.0089 (2)	0.0293 (2)	−0.0005 (2)

### Geometric parameters (Å, °)

**Table:**

N1—N2	1.357 (2)	C16—C17	1.375 (2)
N1—C5	1.370 (2)	C16—H16	0.9300
N1—C11	1.408 (2)	C17—H17	0.9300
N2—C3	1.327 (2)	C31—C36	1.391 (2)
C3—C4	1.418 (2)	C31—C32	1.398 (2)
C3—C31	1.460 (2)	C32—C33	1.369 (2)
C4—C5	1.346 (3)	C32—H32	0.9300
C4—H4	0.9300	C33—C34	1.384 (2)
C5—H5	0.9300	C33—H33	0.9300
C11—C1A	1.351 (2)	C34—C35	1.382 (2)
C11—C12	1.474 (2)	C34—Cl2	1.7366 (18)
C12—C17	1.392 (2)	C35—C36	1.375 (2)
C12—C13	1.399 (2)	C35—H35	0.9300
C13—C14	1.374 (2)	C36—H36	0.9300
C13—H13	0.9300	C1A—C2A	1.431 (3)
C14—C15	1.384 (2)	C1A—H1A	0.9300
C14—H14	0.9300	C2A—O1	1.219 (2)
C15—C16	1.387 (2)	C2A—H2A	1.07 (2)
C15—Cl1	1.7313 (17)		
			
N2—N1—C5	111.32 (14)	C17—C16—H16	120.5
N2—N1—C11	120.54 (13)	C15—C16—H16	120.5
C5—N1—C11	127.88 (15)	C16—C17—C12	121.30 (13)
C3—N2—N1	105.24 (13)	C16—C17—H17	119.3
N2—C3—C4	110.62 (16)	C12—C17—H17	119.3
N2—C3—C31	120.42 (14)	C36—C31—C32	117.70 (16)
C4—C3—C31	128.96 (15)	C36—C31—C3	121.53 (14)
C5—C4—C3	105.75 (15)	C32—C31—C3	120.75 (14)
C5—C4—H4	127.1	C33—C32—C31	121.34 (15)
C3—C4—H4	127.1	C33—C32—H32	119.3
C4—C5—N1	107.06 (16)	C31—C32—H32	119.3
C4—C5—H5	126.5	C32—C33—C34	119.49 (15)
N1—C5—H5	126.5	C32—C33—H33	120.3
C1A—C11—N1	119.59 (16)	C34—C33—H33	120.3
C1A—C11—C12	125.45 (15)	C35—C34—C33	120.66 (16)
N1—C11—C12	114.95 (13)	C35—C34—Cl2	120.04 (13)
C17—C12—C13	118.47 (15)	C33—C34—Cl2	119.29 (13)
C17—C12—C11	120.65 (13)	C36—C35—C34	119.15 (15)
C13—C12—C11	120.88 (14)	C36—C35—H35	120.4
C14—C13—C12	120.84 (15)	C34—C35—H35	120.4
C14—C13—H13	119.6	C35—C36—C31	121.60 (15)
C12—C13—H13	119.6	C35—C36—H36	119.2
C13—C14—C15	119.37 (14)	C31—C36—H36	119.2
C13—C14—H14	120.3	C11—C1A—C2A	123.32 (17)
C15—C14—H14	120.3	C11—C1A—H1A	118.3
C14—C15—C16	121.07 (15)	C2A—C1A—H1A	118.3
C14—C15—Cl1	119.74 (12)	O1—C2A—C1A	123.47 (19)
C16—C15—Cl1	119.20 (13)	O1—C2A—H2A	119.7 (13)
C17—C16—C15	118.93 (15)	C1A—C2A—H2A	116.7 (13)
			
C5—N1—N2—C3	0.51 (16)	C14—C15—C16—C17	−1.3 (2)
C11—N1—N2—C3	175.14 (12)	Cl1—C15—C16—C17	178.47 (11)
N1—N2—C3—C4	−0.49 (16)	C15—C16—C17—C12	0.5 (2)
N1—N2—C3—C31	179.66 (12)	C13—C12—C17—C16	0.7 (2)
N2—C3—C4—C5	0.30 (18)	C11—C12—C17—C16	−178.87 (13)
C31—C3—C4—C5	−179.86 (14)	N2—C3—C31—C36	175.27 (13)
C3—C4—C5—N1	0.03 (18)	C4—C3—C31—C36	−4.6 (2)
N2—N1—C5—C4	−0.34 (18)	N2—C3—C31—C32	−3.1 (2)
C11—N1—C5—C4	−174.48 (14)	C4—C3—C31—C32	177.04 (15)
N2—N1—C11—C1A	−16.7 (2)	C36—C31—C32—C33	−2.1 (2)
C5—N1—C11—C1A	156.99 (16)	C3—C31—C32—C33	176.38 (14)
N2—N1—C11—C12	164.21 (12)	C31—C32—C33—C34	0.4 (2)
C5—N1—C11—C12	−22.1 (2)	C32—C33—C34—C35	1.9 (2)
C1A—C11—C12—C17	−52.2 (2)	C32—C33—C34—Cl2	−176.72 (12)
N1—C11—C12—C17	126.86 (15)	C33—C34—C35—C36	−2.4 (2)
C1A—C11—C12—C13	128.27 (18)	Cl2—C34—C35—C36	176.17 (11)
N1—C11—C12—C13	−52.66 (19)	C34—C35—C36—C31	0.7 (2)
C17—C12—C13—C14	−1.0 (2)	C32—C31—C36—C35	1.5 (2)
C11—C12—C13—C14	178.48 (14)	C3—C31—C36—C35	−176.91 (13)
C12—C13—C14—C15	0.3 (2)	N1—C11—C1A—C2A	173.32 (15)
C13—C14—C15—C16	0.9 (2)	C12—C11—C1A—C2A	−7.7 (3)
C13—C14—C15—Cl1	−178.85 (12)	C11—C1A—C2A—O1	170.98 (18)

### Hydrogen-bond geometry (Å, °)

**Table:**

Cg1 is the centroid of the C31–C36 ring.

**Table:**

*D*—H···*A*	*D*—H	H···*A*	*D*···*A*	*D*—H···*A*
C13—H13···Cl2^i^	0.93	2.93	3.6447 (16)	134
C14—H14···O1^ii^	0.93	2.49	3.407 (2)	167
C36—H36···Cl1^iii^	0.93	2.90	3.6334 (16)	137
C17—H17···Cg1^iv^	0.93	2.68	3.4919 (18)	147

Symmetry codes: (i) −*x*, −*y*, −*z*+1; (ii) *x*−1, *y*, *z*; (iii) −*x*, −*y*, −*z*+2; (iv) −*x*+1, −*y*, −*z*+2.
